# Multiniche mycobiome profiling identifies distinctive fungal dysbiosis in common variable immunodeficiency

**DOI:** 10.3389/fimmu.2026.1804724

**Published:** 2026-05-20

**Authors:** Marta Dafne Cabanero-Navalon, Miguel Carda-Diéguez, Alex Mira, Pedro Moral Moral, Maria Mercedes Diaz Luna, Héctor Balastegui-Martín, Miguel Salavert Lletí, Victor Garcia-Bustos

**Affiliations:** 1 Primary Immune Deficiencies Unit, Department of Internal Medicine, University and Polytechnic Hospital La Fe, Valencia, Spain; 2 Research Group of Chronic Diseases and HIV Infection, Health Research Institute La Fe, Valencia, Spain; 3 Genomics & Health Department, Fundación para el Fomento de la Investigación Sanitaria y Biomédica de la Comunitat Valenciana (FISABIO) Foundation, Valencia, Spain; 4 CIBER Center for Epidemiology and Public Health (CIBER-ESP), Madrid, Spain; 5 Severe Infection Research Group, Health Research Institute La Fe, Valencia, Spain; 6 Unit of Infectious Diseases, University and Polytechnic Hospital La Fe, Valencia, Spain; 7 University Institute of Animal Health and Food Safety (IUSA), University of Las Palmas de Gran Canaria, Arucas, Spain

**Keywords:** biomarkers, *Candida albicans*, common variable immunodeficiency, dysbiosis, fungal microbiota, immune dysregulation, ITS amplicon sequencing, mucosal niches

## Abstract

**Background:**

Common variable immunodeficiency (CVID) is associated with bacterial dysbiosis, particularly in patients with immune dysregulation, but the contribution of the fungal microbiome (mycobiome) remains poorly understood.

**Methods:**

We conducted a cross-sectional, multi-compartment study in 41 adults with CVID (24 with immune dysregulation, dCVID; 17 with infectious-only manifestations, iCVID) and 15 matched healthy controls. Saliva, sputum and stool samples were analyzed using ITS1 amplicon sequencing with amplicon sequence variant-based taxonomic assignment, followed by α/β-diversity analyses, multivariate modeling, differential abundance testing and machine learning approaches for biomarker identification.

**Results:**

Across all three niches, mycobiome composition differed significantly between CVID and controls, whereas dCVID and iCVID did not separate. Fungal richness and evenness were reduced in CVID, most prominently in respiratory and oral samples. ANCOM-BC revealed a reproducible “*Candida*-skewed” configuration in both phenotypes, with marked enrichment of *Candida albicans* in sputum, stool and saliva, accompanied by increased abundance of other opportunistic yeasts such as *Nakaseomyces glabratus*. In contrast, environmental or putatively commensal taxa were consistently depleted. Random forest models based on fungal profiles accurately discriminated CVID from controls, with AUC up to 0.96 (95% CI 0.91-0.99) in saliva and 0.94 (95% CI 0.88-0.99) in stool, whereas classification of dCVID versus iCVID was modest.

**Conclusion:**

Together, these findings provide the first integrated view of mycobiome alterations across multiple ecological niches in CVID, highlighting consistent enrichment of opportunistic yeasts over commensals. The expansion of *C. albicans* supports a potential pathobiont role, and the strong discriminatory performance of fungal signatures underscores their promise as non-invasive biomarkers in this immunodeficiency.

## 1 Introduction

Common variable immunodeficiency (CVID) is the most prevalent symptomatic inborn error of immunity (IEI), characterized by hypogammaglobulinemia, impaired vaccine responses, and remarkable clinical heterogeneity (1). Although immunoglobulin replacement therapy (IgRT) has substantially reduced infectious burden, non-infectious complications, such as autoimmunity, enteropathy, lymphoproliferation and neoplasia, are the main determinants of morbidity and mortality (2). The etiology of CVID remains incompletely understood. While monogenic variants account for up to 30% of cases (3, 4), current evidence supports a complex interplay between genetic predisposition and environmental influences, including microbiota disruption (5, 6).

In recent years, the microbiome has emerged as a key modulator of immune homeostasis and a potential source of biomarkers in immune-mediated diseases. In CVID, bacterial microbiome studies have consistently reported microbiota alterations. Jørgensen et al. (7) demonstrated reduced gut bacterial diversity and increased dysbiosis, correlating with elevated serum levels of lipopolysaccharide, soluble CD14, and IL-2, particularly in patients with immune dysregulation. Furthermore, a gut–lung axis has been proposed, linking intestinal microbial perturbations to systemic and pulmonary immune alterations (8, 9), in line with emerging evidence that the pulmonary microbiota may trigger autoimmune processes (10, 11). Additionally, our group recently characterized the oropharyngeal, respiratory, and fecal bacterial microbiota of CVID patients, identifying phenotype-specific microbial profiles that distinguished those with immune dysregulation from patients with purely infectious manifestations and from healthy controls (12). Similar alterations were also documented in patients with granulomatous-lymphocytic interstitial lung disease (GLILD) (13).

However, the microbiota is not composed solely of bacteria; fungi also represent an integral yet under-studied component. In other immune-mediated diseases, such as HIV infection and inflammatory bowel disease, the fungal microbiome or mycobiome, and especially *Candida* species, has been shown to interact with the immune system, influence inflammation, and modulate immune responses (14–17). Furthermore, several studies have reported Th17 cell abnormalities in subsets of patients with CVID, suggesting impaired antifungal immunity and a potential imbalance in fungal immune control (18, 19). To date, only one study has examined the gut mycobiome in CVID, reporting no significant differences compared with healthy controls (20). However, its exclusive focus on fecal samples, lack of clinical stratification between infectious-only and immune dysregulation phenotypes, and modest sample size may have limited the detection of disease-associated fungal signals. As a result, the contribution of the mycobiome to CVID pathogenesis remains poorly defined.

Therefore, understanding fungal community alterations in patients with CVID compared to healthy individuals, as well as its association with immune dysregulation phenotypes may provide novel insights into disease mechanisms and help identify early, non-invasive biomarkers. This study aimed to analyze the salivary, sputum, and fecal mycobiome in CVID and to evaluate its potential role in phenotypic stratification and clinical outcomes.

## 2 Materials and methods

### 2.1 Patients

**Figure 1:**
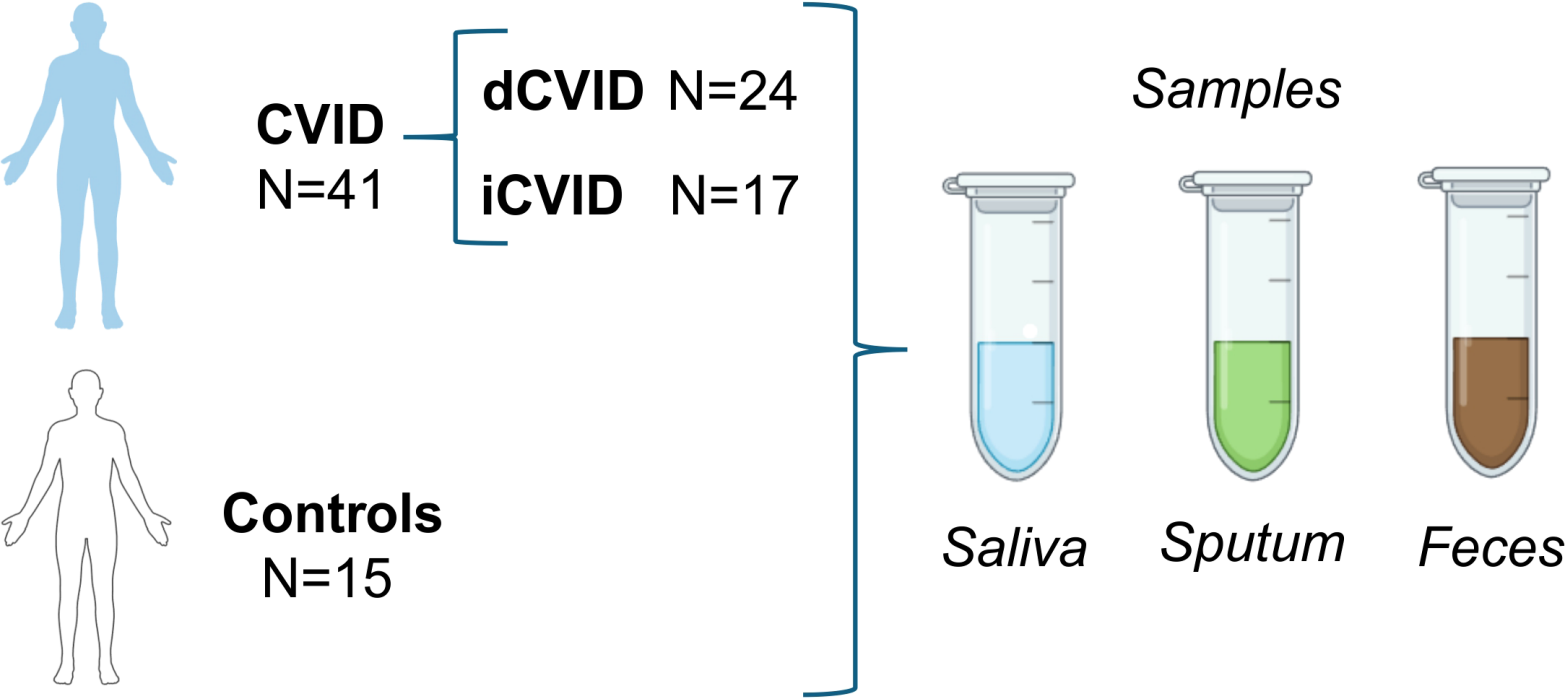
Sampling scheme of CVID and healthy controls. Flow diagram summarizing participant inclusion and sample collection across study groups. A total of 41 adults with CVID (24 with immune dysregulation and 17 with infectious-only manifestations) and 15 matched healthy controls each provided saliva, sputum, and stool specimens for ITS1-based mycobiome profiling.

We conducted a cross-sectional study at the Primary Immunodeficiencies Unit, Department of Internal Medicine, University and Polytechnic Hospital La Fe (Valencia, Spain). Adults (≥18 years) with CVID fulfilling the European Society for Immunodeficiencies (ESID) registry working definitions were eligible; 41 patients under follow-up met inclusion criteria (**Figure 1**). The patient and control cohort analyzed in this study corresponds to the same individuals previously characterized in the bacterial microbiome study by Cabanero-Navalón et al. (12). Demographic, clinical, and laboratory data were abstracted by retrospective chart review. Clinical variables comprised type 1 diabetes mellitus; autoimmune hemolytic anemia (AHA); immune thrombocytopenic purpura (ITP); Evans syndrome; non-infectious generalized lymphadenopathy on chest CT and/or PET-CT; granulomatous-lymphocytic interstitial lung disease (GLILD) confirmed by lung biopsy; splenomegaly on abdominal ultrasound or CT; hepatopathy (elevated liver enzymes, abnormal abdominal imaging, or portal hypertension); non-infectious chronic enteropathy (chronic diarrhea or abnormal gastrointestinal biopsy); and solid or hematologic malignancy. Laboratory variables included serum IgG, IgA, and IgM (mg/dL); CD4+ and CD8+ T-cell counts, CD19+ B-cell counts, natural killer (NK) cell counts (cells/µL); and CD4/CD8 ratio.

Following Chapel et al. (21), patients were classified as CVID with immune dysregulation (dCVID) if any of the following features were present (AHA, ITP, Evans, GLILD, non-infectious lymphadenopathy, hepatopathy, splenomegaly, non-infectious chronic enteropathy, and/or malignancy) or as infectious-only CVID (iCVID) if infections were the sole manifestations. The study was approved by the Ethics Committee of the Health Research Institute La Fe (registry 2020-376-1) and conducted in accordance with the Declaration of Helsinki.

### 2.2 Sampling

Samples were obtained from 41 CVID patients and age- and sex-paired 15 healthy donors as controls, corresponding to the same individuals included in the previous bacterial microbiota study by Cabanero-Navalón et al., 2023. Controls had no active caries or periodontal disease. Individuals who received systemic antibiotics in the prior month were excluded, except for cotrimoxazole or azithromycin when used as prophylaxis; healthy donors were excluded if they had taken any antibiotic in the preceding month. From each participant, one saliva, one sputum, and one stool specimen were collected (**Figure 1**). Unstimulated saliva (~1 mL) was obtained after 3 minutes of drooling; sputum (~1 mL) after deep breathing and forceful coughing. Saliva and sputum were centrifuged, and microbial pellets were stored at −80 °C. Stool (~5 mL) was deposited into a flask containing 5 mL RNAlater^®^ and kept at room temperature until delivery to the laboratory, then stored at −80 °C until DNA extraction.

### 2.3 DNA extraction and sequencing

DNA was extracted using the MagNa Pure LC DNA Isolation Kit II on a MagNa Pure instrument (Roche). Samples underwent ultrasound treatment in a Raypa VCI-50 sonicator bath, at low ultrasound intensity (3 × 10-s cycles) to facilitate disaggregation, enzymatic digestion with zymolase (0.25 mg/ml), lysostaphin (5 kU/mL), lysozyme (100 mg/mL), and mutanolysin (2.5 kU/mL) following Boix-Amoros et al., 2017 (22). Additionally, this mix was incubated 15 min at 65 °C with 20 µl of 1% Glucanex in PBS for additional lysis of fungal β-glucans. This was, followed by Proteinase K treatment. DNA concentration was quantified using the Quant-iT™ PicoGreen^®^ dsDNA Assay and a Qubit™ 3 Fluorometer (Thermo Fisher Scientific).

Primers targeting the fungal internal transcribed spacer 1 (ITS1) region of the rRNA operon (forward: TAGAGGAAGTAAAAGTCGTAA; reverse: TTYRCTRCGTTCTTCATC; IUPAC degeneracies) were used (23), with Illumina adapter overhangs, for sequencing on an Illumina MiSeq platform. Sequencing was performed at the Foundation for the Promotion of Health and Biomedical Research (FISABIO, Valencia, Spain). No-template PCR controls and extraction blanks were included and processed in parallel to monitor contamination during DNA extraction, library preparation, and sequencing. Control samples showed no signs of contamination. Raw reads have been deposited in NCBI SRA public database (Bioproject: PRJNA1374249).

### 2.4 Data bioinformatic analysis

Paired-end ITS1 reads were merged with FLASH (24), quality-trimmed in sliding windows and length-filtered using PRINSEQ-lite (25), and screened for chimeras with UCHIME (26). Amplicon sequence variants (ASVs) were then inferred with DADA2 (27), and taxonomic assignment was performed using the stand-alone RDP Classifier trained on the UNITE fungal ITS reference database (28). The ASV table was rarefied to an even sequencing depth prior to diversity analyses. Alpha diversity (Shannon, Chao1) were computed in R (with vegan and plyr). Clinical characteristics were compared with Fisher’s exact tests and ANOVA after assessing normality (Q–Q plots) and homoscedasticity (Levene’s test). An Adonis test (Permutational Multivariate Analysis of Variance Using Distance Matrices), provided by the Vegan library of R was used for performing multivariate analyses. Rarefaction curves and alpha diversity indexes (Chao1, Pielou’s evenness, dominance [dbp] and Shannon) were calculated using the minimum number of annotated reads (10,000). Differential abundance was tested with ANCOM-BC (Analysis of Compositions of Microbiomes with Bias Correction) on raw ASV count table (29). Prior to analysis, low-prevalence and low-abundance taxa were filtered to reduce noise and avoid sparse features. Specifically, taxa were retained only if their relative abundance exceeded a minimum threshold defined as ten times the smallest non-zero relative abundance observed in the dataset, and if this threshold was reached in at least 60% of samples within at least one study group. This filtering procedure removed extremely rare taxa while retaining consistently detected features suitable for compositional differential abundance testing. Because ANCOM-BC2 was applied to pairwise group comparisons, prevalence and abundance filtering was performed independently for each comparison. After filtering, the number of retained features ranged from 15–17 for stool samples, 20 for saliva samples, and 17–20 for sputum samples.

Discriminatory fungal biomarkers were prioritized using random forest models with Boruta feature selection (30). To reduce dimensionality, the analysis was restricted to taxa showing significant differential abundance between groups (p ≤ 0.005) in univariate tests. Feature importance was evaluated using the Boruta algorithm with 500 iterations and 1,000 trees per random forest, and features classified as “confirmed” were retained. For model evaluation, stratified random train–test splits were performed, ensuring that class proportions were preserved between training and test sets. In each iteration, 50% of samples were used for training and 50% for testing, and a random forest model (1,000 trees) was trained on the selected features. To obtain robust estimates of model performance and associated uncertainty, the area under the receiver operating characteristic curve (AUC) was calculated using bootstrap resampling (5,000 replicates), from which 95% confidence intervals were derived. Because of class imbalance between CVID patients and healthy controls, additional performance metrics were computed, including balanced accuracy, sensitivity, specificity, precision and F1 score. Out-of-bag (OOB) error estimates were also examined as an internal measure of model stability. Given the relatively small sample size, this analysis was intended for exploratory biomarker prioritization rather than for building a fully independent predictive model. All machine learning analyses were implemented in R using the packages randomForest, Boruta, and pROC.

Finally, the abundance of bacteria based on 16S rRNA sequencing data from our previous publication (12) and the abundance of fungi obtained in the current work were correlated using sPLS canonical method from mixOmics package (31). Both bacteria and fungi abundances were adjusted using ANCOMBC2 approach to account for the compositional nature of microbiome datasets. A pseudocount of 1 was added prior to transformation to handle zero values. Pairwise associations were then assessed using Spearman’s rank correlation coefficient. To further reduce the likelihood of spurious correlations inherent to compositional data and to capture multivariate relationships, we complemented this approach with sparse Partial Least Squares canonical analysis (sPLS-canonical). Only associations that were consistently supported by both approaches were retained. Specifically, correlations were considered robust when they showed an absolute correlation coefficient from sPLS-canonical (|r|) > 0.5 and a Spearman correlation with p.value < 0.05. This combined strategy was used to identify stable and biologically meaningful cross-domain interactions.

## 3 Results

### 3.1 Population

From a total of 41 CVID patients, 24 fulfilled criteria of dCVID and 17 had only suffered infectious complications (iCVID), as in 12 (**Supplementary Table 1**). Mean (SD) age was 43.9 (15.9) years in dCVID, 49.9 (16.9) in iCVID, and 44.4 (14.2) in healthy controls. Females comprised 12/24 (50.0%) in dCVID, 12/17 (70.6%) in iCVID, and 9/15 (60.0%) in controls; age and sex distributions were comparable across groups (p=0.1). In dCVID patients, ITP occurred 10/24 (41.7%), Evans’ syndrome 5/24 (20.8%), and AHA in 5/24 (20.8%). Non-infectious generalized lymphadenopathy was present in 10/24 (41.7%); splenomegaly in 12/24 (50.0%); systemic autoimmune disease in 5/24 (20.8%); chronic enteropathy in 11/24 (45.8%); and GLILD was histologically confirmed in 7/24 (29.2%). Malignancy (solid or hematologic) was observed in 3/24 (12.5%). The iCVID group showed no immune-dysregulation complications; one participant had lymphadenopathy of infectious etiology (1/17, 5.9%), and there were no cases of splenomegaly, systemic autoimmunity, enteropathy, GLILD, or malignancy (0/17 for each). Healthy controls had none of these conditions (0/15 for each). Lung disease burden was higher in dCVID, with a Baumann score of 8.02 (8.48) versus 1.33 (1.63) in iCVID. Immunophenotyping (mean [SD], cells/µL) showed CD4+ counts of 581.2 (487.9) in dCVID and 662.9 (248.1) in iCVID; CD8+ counts of 458.0 (355.6) and 563.8 (307.8), respectively. Serum IgA (mg/dL) averaged 26.33 (42.41) in dCVID and 43.94 (44.44) in iCVID.

### 3.2 Global multivariate compositional differences in the mycobiome by clinical group

In order to study the mycobial composition of sputum, saliva and fecal samples we sequenced the ITS hypervariable region. A mean of 2×10^5^ reads per sample were obtained which were sufficient to covered most of the diversity (**Supplementary Figure 1**). Using a prevalence threshold of ≥75% across samples, the core mycobiome consisted of six genera shared across niches: *Saccharomyces*, *Candida*, *Penicillium*, *Malassezia*, *Debaryomyces*, and *Cladosporium*. Low levels of *Candida albicans* and *Saccharomyces bayanus* were detected in negative sequencing controls. However, given the very low sequencing depth of these controls (190–508 reads), relative abundance estimates are subject to high stochastic variability and should be interpreted with caution. These taxa were consistently detected at substantially higher prevalence across biological samples, supporting their biological relevance. Nevertheless, their presence in negative controls suggests that low-level background contamination cannot be entirely excluded and should be considered when interpreting results.

**Figure 2:**
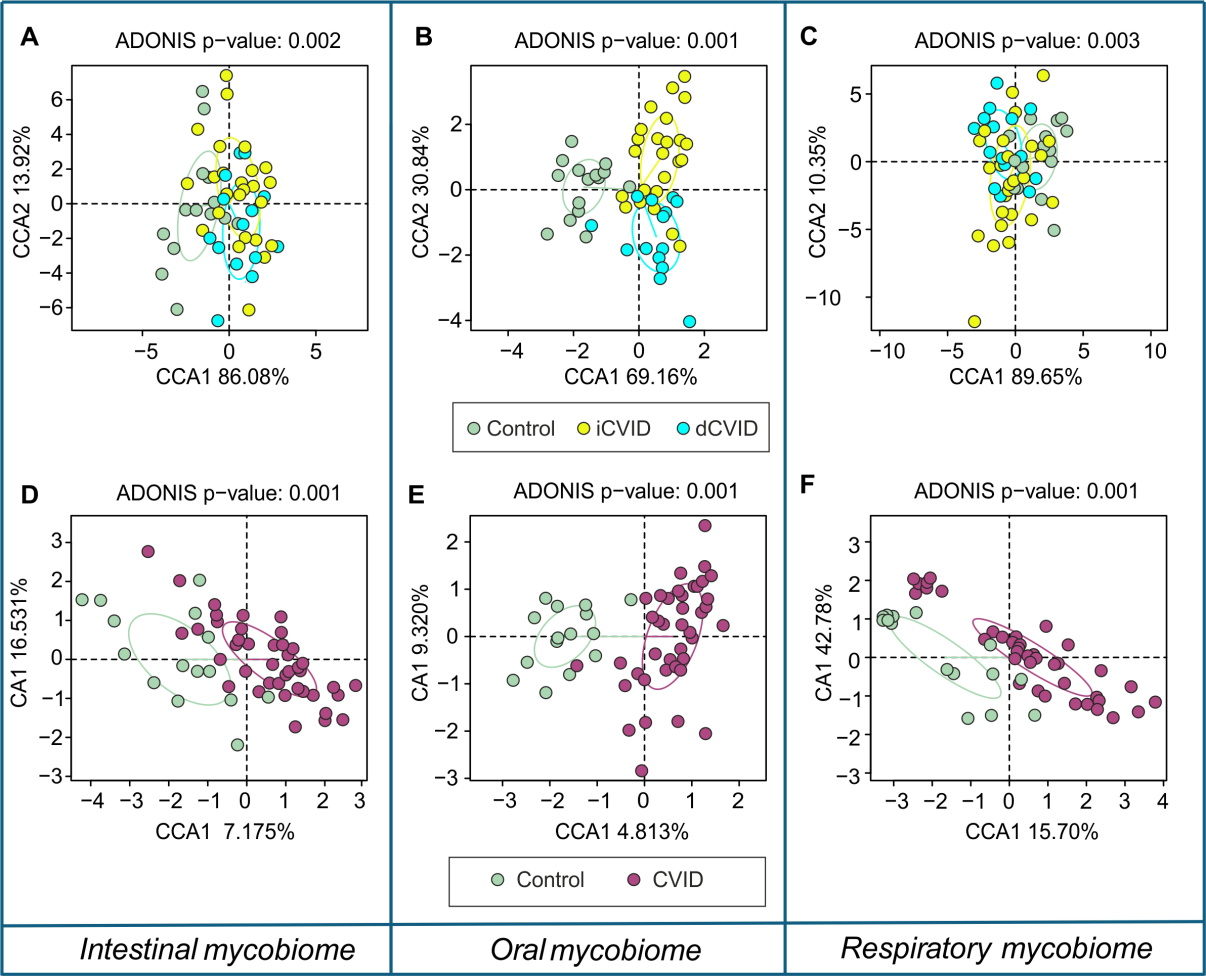
Canonical correspondence analysis (CCA) of fungal community structure across different ecological niches in CVID patients and healthy controls, with ADONIS p-values for overall group separation. **(A–C)** display overall group comparisons, and **(D–F)** depict pairwise contrasts. **(A)** Stool CCA shows significant separation between CVID and healthy controls (ADONIS p = 0.002), with both dCVID and iCVID contributing to this shift. **(B)** Saliva exhibits a similar pattern, with strong global differences (ADONIS p = 0.001) and separation of both CVID phenotypes from controls. **(C)** In sputum, CVID mycobiomes also differ markedly from healthy individuals (ADONIS p = 0.003). **(D–F)** show two-by-two comparisons for each niche. **(D)** Stool: controls differ significantly from CVID (ADONIS p = 0.001), from dCVID (p = 0.005), and from iCVID (p = 0.001), whereas dCVID and iCVID do not differ from each other (p = 0.8). **(E)** Saliva: controls separate clearly from CVID (p = 0.001), dCVID (p = 0.002), and iCVID (p = 0.003), while no separation occurs between dCVID and iCVID (p = 0.23). **(F)** Sputum: controls differ from CVID (p = 0.001), dCVID (p = 0.001), and iCVID (p = 0.009), but again no differences are observed between the two CVID phenotypes (p = 0.13).

In sputum, an additional genus (*Aspergillus*) reached this prevalence threshold. High interindividual variation was observed in all three sample types when the proportion of these six genera were observed in each sample (**Supplementary Figure 2**). Across sputum, stool, and saliva, multivariate analyses demonstrated consistent fungal compositional shifts in CVID compared with healthy controls, affecting both dCVID and iCVID, while no significant differences were observed between the two CVID phenotypes (**Figure 2**). In sputum, overall comparisons were significant (ADONIS p = 0.003, R^2^ = 0.06), with clear separation between controls and CVID (ADONIS p = 0.001, R^2^ = 0.04). Both dCVID (ADONIS p = 0.001, R^2^ = 0.06) and iCVID (ADONIS p = 0.009, R^2^ = 0.05) patients differed from controls (**Figure 2F**), while dCVID and iCVID between themselves did not (ADONIS p = 0.13, R^2^ = 0.02) (**Figure 2C**). Stool analyses showed similar results (ADONIS p = 0.002, R^2^ = 0.), with controls differing from CVID (ADONIS p = 0.001, R^2^ = 0.02), dCVID (ADONIS p = 0.005, R^2^ = 0.07), and iCVID (ADONIS p = 0.001, R^2^ = 0.01), but no differences between dCVID and iCVID (ADONIS p = 0.8, R^2^ = 0.03) (**Figures 2A, D**). In saliva, overall differences were again significant (ADONIS p = 0.001, R^2^ = 0.05), with controls differing from CVID (ADONIS p = 0.001, R^2^ = 0.03), dCVID (ADONIS p = 0.002, R^2^ = 0.05), and iCVID (ADONIS p = 0.003, R^2^ = 0.04), whereas dCVID and iCVID did not differ (ADONIS p = 0.23, R^2^ = 0.03) (**Figures 2B, E**).

### 3.3 Indices of alpha diversity

Alpha diversity analyses revealed consistent reductions in microbial richness and evenness in CVID compared with healthy controls, with some variation across sample types (**Supplementary Figure 3**). In sputum, CVID patients showed significantly lower Chao1 richness (p = 4.3×10^-5^) and Pielou’s evenness (p = 0.0028), while Shannon diversity was not significant (p = 0.064) and DBP showed only a borderline difference (p = 0.051). Specifically, dCVID as a complicated phenotype subgroup similarly exhibited reduced richness (Chao1, p = 5.7×10^-5^) and evenness (p = 0.023), but no differences in Shannon (p = 0.41) or DBP (p = 0.54). In stool, richness was significantly decreased in CVID (Chao1, p = 0.0041), whereas Shannon (p = 0.63), evenness (p = 0.27), and DBP (p = 0.94) did not differ; dCVID did not differ from controls in any index (all p > 0.05). In saliva, CVID patients displayed reduced richness (Chao1, p = 0.037) and evenness (p = 0.041), but not Shannon (p = 0.17) or DBP (p = 0.074). dCVID also showed diminished richness (Chao1, p = 0.013), with no differences in Shannon (p = 0.46), evenness (p = 0.11), or DBP (p = 0.29).

### 3.4 Comparative fungal species distribution in CVID phenotypes and controls

**Figure 3:**
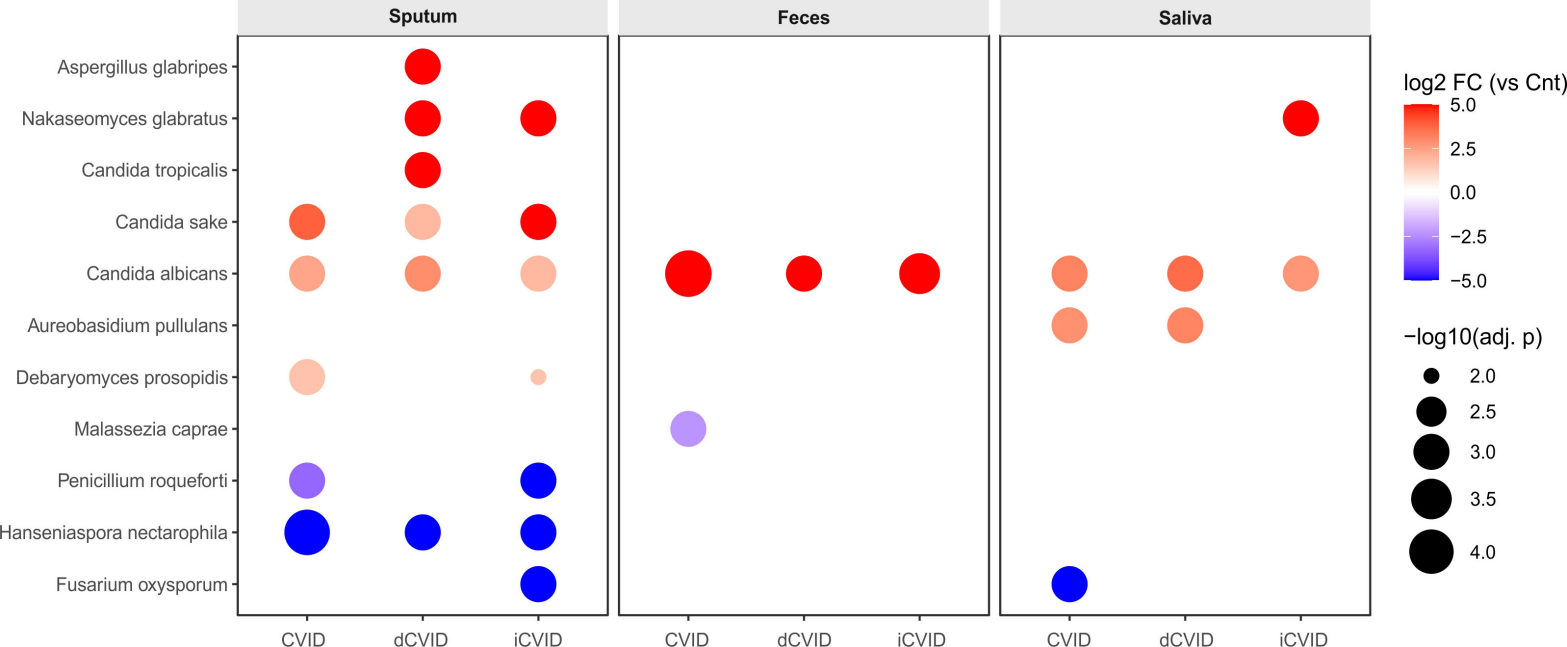
Differentially abundant fungal taxa across mucosal niches and CVID phenotypes. Dot plot showing ANCOM-BC–derived differential abundance of key fungal taxa in respiratory, fecal and salivary samples from patients with common variable immunodeficiency (CVID) compared with healthy controls. Columns represent comparisons of all CVID, immune dysregulation CVID (dCVID) and infectious-only CVID (iCVID) versus healthy controls, and rows correspond to taxa that were significant in at least one contrast. Point color encodes log_2_ fold change in relative abundance (red, enrichment in CVID; blue, depletion), and point size is proportional to –log_10_ of the adjusted p-value.

ANCOM-BC revealed distinct fungal shifts between CVID patients and healthy controls across sample types (**Figure 3**). In sputum, CVID patients showed enrichment of *C. albicans* (8.47% vs. 1.67%, adj. p = 0.03), *Candida sake* (0.44% vs. 0.03%, adj. p = 0.02), and *Debaryomyces prosopidis* (3.93% vs. 1.25%, adj. p = 0.04), with depletion of *Hanseniaspora nectarophila* (0.043% vs. 3.23%, adj. p = 7x10-5) and *Penicillium roqueforti* (0.56% vs. 5.37%, adj. p = 0.01). In stool, *C. albicans* was markedly enriched (3.13% vs. 0.035%, adj. p = 5x10-5) and *Malassezia caprae* reduced (0.15% vs. 0.75%, adj. p = 0.02). In saliva, enrichment of *C. albicans* (9.81% vs. 1.12%, adj. p = 0.01) and *Aureobasidium pullulans* (0.50% vs. 0.07%, adj. p = 0.02) co-occurred with depletion of *Fusarium oxysporum* (0.006% vs. 2.33%, adj. p = 0.01).

When comparing dCVID and iCVID, no statistically significant differences were identified by ANCOM-BC in sputum, stool, or saliva (**Figure 3**). In sputum, several taxa, including *Alternaria eichhorniae*, non-assigned ASV of *Aspergillus* spp., *A. pullulans*, *Cladosporium herbarum*, *C. albicans*, and *Candida parapsilosis*, varied in relative abundance between phenotypes, but none reached significance after correction (all adj. p ≥ 0.46).

Relative to healthy controls, dCVID patients exhibited strong enrichment of *C. albicans* across sputum (12.57% vs. 1.67%, adj. p = 0.006), stool (5.76% vs. 0.03%, adj. p = 0.001), and saliva (13.86% vs. 1.12%, adj. p = 0.01). Additional sputum enrichments included *Nakaseomyces glabratus* (0.17% vs. 0, adj. p = 0.006), *C. sake* (0.11% vs. 0.03%, adj. p = 0.04), *Candida tropicalis* (0.052% vs. 0.0004%, adj. p = 0.04), and *Aspergillus glabripes* (0.89% vs. 0, adj. p = 0.01), with depletion of *H. nectarophila* (0 vs. 3.23%, adj. p = 0.006). In saliva, *A. pullulans* was also enriched (0.60% vs. 0.07%, adj. p = 0.02).

Similarly, iCVID patients showed consistent enrichment of *C. albicans* in sputum (6.1% vs. 1.67%, adj. p = 0.003), stool (1.61% vs. 0.035%, adj. p = 0.0003), and saliva (7.39% vs. 1.12%, adj. p = 0.001). Other enriched taxa included *N. glabratus* (0.44% vs. 0, adj. p = 0.003 in sputum; 0.59% vs. 0, adj. p = 0.006 in saliva), *C. sake* (0.63% vs. 0.003%, adj. p = 0.03 in sputum), and *D. prosopidis* (3.9% vs. 1.25%, adj. p = 0.05 in sputum). In contrast, *H. nectarophila* (0.067% vs. 3.23%, adj. p = 0.003), P. roqueforti (0.16% vs. 5.37%, adj. p = 0.01), and *F. oxysporum* (0.004% vs. 2.33%, adj. p = 0.001) were significantly depleted in iCVID compared with controls.

### 3.5 Discriminatory mycobiome signatures and classification performance

**Figure 4:**
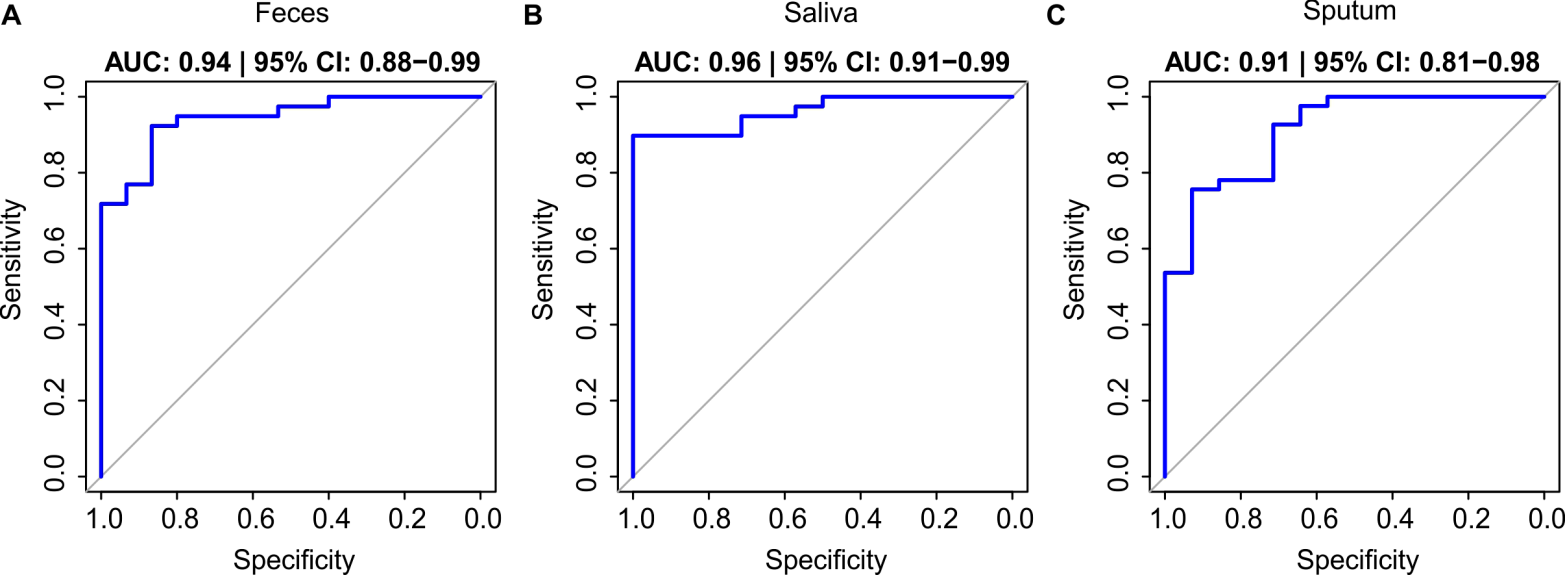
Receiver operating characteristic (ROC) curves of random forest models discriminating CVID patients from healthy controls across different sample types. Random forest classifiers were trained using Boruta-selected fungal taxa to distinguish CVID patients from healthy controls in **(A)** stool, **(B)** saliva and **(C)** sputum samples. Model performance was evaluated using stratified train–test splits. The area under the ROC curve (AUC) with 95% confidence intervals (CI), estimated by bootstrap resampling (5,000 replicates), was as follows: stool (AUC = 0.94, 95% CI: 0.88–0.99), saliva (AUC = 0.96, 95% CI: 0.91–0.99), and sputum (AUC = 0.91, 95% CI: 0.81–0.98).

Due to the shared differences among both CVID clinical phenotypes against controls, Boruta analysis was performed to find a fungal signature as a biomarker of disease. Random forest classifiers based on fungal profiles showed moderate performance in stool (AUC = 0.94, 95% CI: 0.88–0.99, balanced accuracy = 0.81) (**Figure 4A**), good discriminatory performance for CVID versus controls in saliva (AUC = 0.96, 95% confidence interval (CI): 0.91–0.99, balanced accuracy = 0.92) (**Figure 4B**), and lower performance in sputum (AUC = 0.91, 95% CI: 0.81–0.98, balanced accuracy = 0.75) (**Figure 4C**, **Supplementary Table 2**).

In stool, the six discriminatory species of the model included *C. albicans*, *Candida dubliniensis* (0.15% vs. 0.025%), *M. caprae* (0.15% vs. 0.75%), *P. roqueforti* (4.20% vs. 1.08%), *C. herbarum* (1.03% vs. 0.38%), and *S. bayanus* (3.60% vs. 2.44%). In saliva, the model was based on the combination of *F. oxysporum*, *C. albicans*, *N. glabratus*, *Sungouiella intermedia/pseudointermedia* (3.10% vs. 1.35%), and *A. pullulans*. The sputum-based model was driven by *C. albicans*, *P. roqueforti*, and *M. restricta* (1.42% in CVID vs. 4.14% in controls).

When comparing dCVID and iCVID directly, Boruta models reached only modest predictive performance (AUC = 0.77). Six species contributed, including *D. prosopidis* (2.86% in iCVID vs. 0.99% in dCVID), *Aspergillus ruber* (1.46% vs. 3.04%), an unassigned ASV of *Alternaria* spp. (1.60% vs. 3.22%), *C. herbarum* (0.55% vs. 0.99%), *Malassezia arunalokei* (2.38% vs. 3.28%), and an additional *D. prosopidis* variant (1.74% vs. 3.81%).

In dCVID versus controls, sputum yielded an AUC of 0.83, with four discriminatory taxa: *C. albicans*, *C. herbarum* (0.94% vs. 2.09%), *P. roqueforti* (1.27% vs. 5.37%), and *S. bayanus* (2.20% vs. 1.86%). Stool achieved higher predictive accuracy (AUC = 0.90), with *C. albicans*, *C. dubliniensis* (0.10% vs. 0.025%), *M. caprae* (0.13% vs. 0.75%), *S. bayanus* (0.063% vs. 0.46%), and *C. parapsilosis* (0.09% vs. 0.005%). In saliva, the model also reached an AUC of 0.90, based on *C. albicans*, *A. pullulans*, *P. roqueforti* (0.046% vs. 0.057%), *F. oxysporum*, and an unassigned ASV of *Metschnikowia* sp. (2.92% vs. 1.35%).

For iCVID versus controls, Boruta selected three taxa in sputum, *P. roqueforti*, *M. restricta* (1.58% vs. 4.14%), and *C. albicans*, yielding an AUC of 0.79. Stool models performed strongly (AUC = 0.91), with six discriminatory taxa: *C. albicans*, *C. dubliniensis* (0.18% vs. 0.025%), *P. roqueforti* (6.53% vs. 1.08%), *S. bayanus* (3.07% vs. 2.44%), *Geotrichum silvicola* (1.01% vs. 6.20%), and *M. caprae* (0.16% vs. 0.75%). Saliva achieved the highest performance (AUC = 0.92), with six predictive species, including *F. oxysporum*, *N. glabratus*, *M. restricta* (3.36% vs. 6.08%), *S. intermedia/pseudointermedia* (3.05% vs. 1.35%), and two distinct *C. albicans* strains with different ASVs (7.39% vs. 1.12% and 7.66% vs. 8.03%).

### 3.6 Bacterial-fungal correlations

The correlation between the abundance of bacteria and fungi in sputum, stool and saliva samples for controls or CVID patients has been assessed. Regardless of the type of sample, the correlations detected in healthy controls were different than those observed in CVID patients. In control sputum samples, *S. bayanus*, *P. roqueforti* or *D. prosopidis* correlated positively with *Veillonella* and *Prevotella* species, *Fusobacterium periodonticum* among others; and negatively with *Tannerella forsythia*, unclassified *Rikenellaceae RC9* and *Filifactor alocis* (**Supplementary Figure 4**). In addition, *M. caprae* positively correlated with *Corynebacterium matruchotii*, *Saccharimonadaceae* and *Campylobacter concisus.* Interestingly, in CVID sputum samples the fungi *A. pullulans* correlated positively with *T*. *forsythia*, *Fusobacterium nucleatum* and *Saccharimonadaceae.*

Similarly, in stool samples we detected different correlations depending on whether we analyze control or CVID samples. In control patients, *C. herbarum* and *M. caprae* correlated positively with *Ruminococcus gnavus* whereas correlated negatively with *Lachnospiraceae*, *Oscillibacter*, *Oscillospira*, *Oscillospirales UCG-005*, *Oscillospirales UCG*-*010* and *Oscillospirales NK4A214* (**Supplementary Figure 5**). *P. roqueforti* correlated negatively with *Blautia obeum*, *Escherichia-Shigella* species and *Barnesiella intestinihominis*. Meanwhile, in CVID patients, *S. bayanus* correlated positively with *Streptococcus cristatus* and other *Streptococcus* species, *Romboutsia* and *Gemella sanguinis*; and negatively with *Oscillibacter valericigenes*.

Finally, in saliva samples the number of bacterial species correlating significantly increased considerably. First of all, *N. glabratus* and *S. bayanus* positively correlated with *F. periodonticum*, *Stomatobaculum saburreum*, *Haemophilus*, *Alloprevotella* unclassified, *Prevotella pallens*, *Prevotella melaninogenica*, *Veillonella* unclassified and *V*. *rogosae*, *Atopobium parvulum*, *Leptotrichia wadei* and unclassified and *Eubacterium nodatum* (**Supplementary Figure 6**). Interestingly, in saliva samples of CVID patients some of these bacterial species (*P. pallens*, *P. melaninogenica*, *Veillonella atypica*) also correlated positively but with a different fungi species: *C. parapsilosis*.

## 4 Discussion

This study demonstrates that the fungal microbiota is significantly altered in CVID compared with healthy controls across multiple ecological compartments. This comprehensive study of saliva, sputum, and stool samples revealed consistent compositional shifts and reduced fungal richness and evenness, independently on the immune dysregulation phenotype of the patients. At the taxonomic level, CVID was characterized by significant enrichment of pathogenic and other opportunistic fungi, such as *C. albicans*, coupled with depletion of environmental and commensal taxa in comparison with healthy individuals, which was homogenous in the whole CVID population. These alterations were reproducible across all three biological niches. Importantly, machine learning approaches identified discriminatory fungal signatures that reliably distinguished CVID patients from controls, achieving excellent classification performance (AUC up to 0.96), whereas differences between infectious-only and immune dysregulation clinical phenotypes were modest.

These results contrast with the limited evidence previously available on the mycobiome in CVID, where no significant alterations in the gut fungal microbiota have previously been reported (20). This could be caused by many factors including the differences in the primers used by Fiedorova and the ones used in this study. In contrast, an expanding body of research on the bacterial microbiota in CVID has consistently demonstrated marked differences between clinical phenotypes, from the milder predominantly infectious phenotype to the more severe immune dysregulation phenotype, both in overall biodiversity indices and in the presence of specific bacterial taxa (5, 7, 12, 32, 33). These bacterial community shifts have also been assessed across the same three compartments, namely the oropharyngeal, respiratory and gut microbiota, reinforcing the compartment-specific relevance of microbial alterations in CVID (12).

Despite growing interest in microbiome-driven inflammation and autoimmunity in CVID, the mycobiome remains largely unexplored, with only one prior study in 27 non-phenotyped patients, restricted to stool and household-matched controls (20). That work reported low diversity, high inter-individual variability and only sporadic genus-level associations, suggesting a limited role for fungi in CVID pathogenesis, but its gut-only focus, absence of clinical stratification and dominant household-related signal may have obscured disease-specific patterns. By extending sampling to saliva and sputum in addition to stool, and the stratification of patients by phenotype, we identified niche-specific shifts and a consistent enrichment of pathogenic and opportunistic fungi over commensals, in line with a proposed gut–lung axis in CVID (9). Methodologically, our use of ASV-based ITS profiling with species-level assignment and updated statistical approaches, rather than OTU clustering with stringent rare-taxon filtering, may further explain why we detected reproducible CVID-associated mycobiome alterations where the earlier study suggested predominantly stochastic distributions.

Beyond documenting global shifts in fungal community structure, our findings suggest that *C. albicans* could behave as a potential pathobiont in CVID. We observed marked and reproducible enrichment of *C. albicans* in respiratory, fecal and salivary samples in both dCVID and iCVID, together with increased abundance of other opportunistic yeasts such as *N. glabratus* and *C. tropicalis*. Similar *Candida*-dominated mycobiome configurations have been described in HIV infection and inflammatory bowel disease, where expansion of specific *Candida* strains is linked to epithelial barrier disruption, exaggerated Th17 responses and chronic inflammation (14–17). In CVID, hypogammaglobulinaemia, impaired mucosal IgA and defects in Th17/Treg balance (18, 19) may jointly weaken antifungal surveillance, allowing *C. albicans* to overgrow at multiple mucosal sites and potentially act as a driver of persistent immune activation rather than a passive commensal. The relationship between our results and antifungal defense immune mechanisms should be analyzed in further studies.

The broader ecological context of our data is consistent with reports of mycobiome-driven immune dysregulation in immunodeficiencies. In people living with HIV receiving antiretroviral therapy, Gosalbes et al. (14) described a richer but *Candida-* and *Debaryomyces-*enriched gut mycobiome, in which *Candida* spp. correlated positively with circulating IL-17, IL-22 and markers of systemic inflammation, while non-*Candida* molds and yeasts were relatively depleted (14). Li et al. reported similar patterns across mucosal sites in HIV, where loss of Th17/IL-22 responses and epithelial barrier damage favored opportunistic fungal overgrowth and translocation (15). In our CVID cohort, we observe a comparable “*Candida*-skewed” configuration: expansion of *C. albicans*, *N. glabratus* and other opportunistic yeasts, together with depletion of environmental or putatively commensal taxa such as *H. nectarophila*, *P. roqueforti*, *F. oxysporum* and *M. caprae*. Together with experimental evidence that commensal fungi contribute to colonization resistance against *Candida* overgrowth (17), these findings support a mechanistic model in which CVID-associated immune defects and bacterial dysbiosis drive the loss of “protective” fungi and allow *C. albicans* to assume a potential pathobiont role across multiple compartments. It remains to be determined whether the expansion of *C. albicans* in CVID represents a disease-specific phenomenon or a more general feature of inflammatory or immune-mediated disorders.

Our machine learning analyses demonstrated that fungal community profiles can robustly discriminate CVID patients from healthy controls, achieving high predictive accuracy across different mucosal niches. Stool and saliva-based models, in particular, reached AUC values above 0.90, highlighting the reproducibility and strength of these signatures. While the distinction between infectious-only and immune dysregulation phenotypes remained modest, the consistent differences observed between CVID and controls suggest that the mycobiome may harbor disease-associated biomarkers. These findings could raise the possibility of developing non-invasive, mycobiome-based diagnostic tools that might facilitate earlier identification of CVID, particularly in individuals with subtle clinical manifestations, where diagnosis is often delayed (34). Such predictive approaches may complement immunological testing, offering a novel path toward earlier recognition and intervention in this heterogeneous disorder, although their diagnostic and prognostic utility requires validation in larger and longitudinal studies.

Given the limited available data on oral, pulmonary, and gut microbiota and mycobiota, correlations were assessed between fungal abundances identified in this study and bacterial relative abundances reported previously by our group (12) Several of the observed bacteria–fungi associations are consistent with previously reported inter-kingdom interactions. In particular, positive correlations between *Candida*/*Saccharomyces* and anaerobic oral bacteria such as *Veillonella*, *Prevotella* and *Fusobacterium* have been described in oral biofilm models, where *Candida* can promote the growth of strictly anaerobic taxa by modifying the local microenvironment (35). Similarly, associations between *Candida* species and periodontal pathogens such as *Tannerella* have been reported, suggesting complex competitive or cooperative interactions (36). More broadly, the co-dominance of *Candida* and *Malassezia* with bacterial genera such as *Prevotella* and *Veillonella* reflects recurrent ecological configurations in the human oral microbiome (37). Importantly, the marked differences observed between controls and CVID patients suggest a disease-associated rewiring of these inter-kingdom networks.

This study has several limitations that must be acknowledged. First, its cross-sectional design precludes causal inference regarding whether fungal alterations contribute to disease pathogenesis or merely reflect the underlying immune defect. Second, despite including three compartments, the sample size was relatively modest, which may have limited statistical power to detect subtle phenotype-specific associations. Third, the use of ITS amplicon sequencing, while standard for fungal profiling, has intrinsic biases related to primer coverage and taxonomic resolution, which may underestimate certain fungal taxa. Furthermore, the relationship between fungal alterations and clinical or immunological parameters was not specifically addressed and deserves further study. We did not directly characterize Th1/Th17 cell populations or IL-17/IL-22 levels, which limits mechanistic insight into how specific immune pathways shape fungal colonization patterns in CVID. Moreover, potential environmental and dietary influences could not be fully excluded despite stringent inclusion criteria, and sputum samples are prone to contamination by saliva. Finally, the absence of absolute quantification, strain-level resolution and viability assessment limits the interpretation of these findings in terms of true multi-site colonization. Future longitudinal and multi-omics studies integrating bacterial, viral and fungal datasets with detailed immunophenotyping of T-cell subsets and cytokine profiles will be essential to validate and refine these observations and to elucidate the causal links between immune dysfunction and mycobiome disruption in CVID.

## 5 Conclusion

In summary, our study suggests that the mycobiome is altered in CVID across multiple mucosal niches, with a consistent enrichment of opportunistic fungi such as *C. albicans* and depletion of commensal taxa. These findings point toward a potential imbalance in host–fungal interactions in this immunodeficiency. Predictive modeling demonstrated that mycobiome signatures could serve as non-invasive biomarkers of CVID for earlier diagnosis and improved clinical stratification, although these results should be interpreted cautiously and require validation in larger, independent cohorts offering opportunities. These findings broaden the understanding of microbiome dysbiosis in CVID and open new avenues for diagnostic innovation and mechanistic research.

## Data Availability

The original contributions presented in the study are included in the article, sections Material and Methods and Results, as well as in the **Supplementary Material**. Further inquiries can be directed to the corresponding author.
