# Supplementary material for: Multiniche mycobiome profiling identifies distinctive fungal dysbiosis in common variable immunodeficiency
Source: Front Immunol. 2026 May 20;17:1804724. doi: 10.3389/fimmu.2026.1804724 (PMC13229628; doi:10.3389/fimmu.2026.1804724)

Supplementary Material

# Supplementary Tables

**Table S1.** Baseline clinical characteristics of the three study groups.

|  | **Dysimmune-CVID (*n* = 24)** | **Infections-only CVID (*n* = 17)** | **Healthy controls (*n* = 15)** |
| --- | --- | --- | --- |
| Age | 43.87 (15.92) | 49.88 (16.90) | 44.42 (14.19) |
| Females | 50% | 71% | 60% |
| Autoimmune cytopenias |  |  |  |
| ITP | 41.6% | 0 | 0 |
| Evan's syndrome | 21% | 0 | 0 |
| AHA | 21% | 0 | 0 |
| Lymphadenopathies | 41.6% | 0.5% | 0 |
| Splenomegaly | 50% | 0 | 0 |
| Autoimmune systemic disease | 20.8% | 0 | 0 |
| Enteropathy | 45.8% | 0 | 0 |
| Malignancy | 12.5% | 0.5% |  |
| GLILD | 29.1% | 0 | 0 |
| Prophylactic antibiotic use | 29.2% | 11.8% | 0 |
| Immunosuppressant treatment | 37.5% | 5.9% | 0 |

**Table S2.** Predictive performance for common variable immunodeficiency (CVID) of a random forest model based on combination of fungal taxa across sample types. AUC: Area Under the Receiver Operating Characteristic (ROC) curve. F1: F1 precision-recall score.

| **Niche** | **AUC** | **Balanced accuracy** | **Sensitivity** | **Specificity** | **F1** |
| --- | --- | --- | --- | --- | --- |
| Saliva | 0.96 | 0.92 | 0.97 | 0.86 | 0.96 |
| Stool | 0.94 | 0.81 | 0.95 | 0.67 | 0.91 |
| Sputum | 0.91 | 0.75 | 0.93 | 0.57 | 0.89 |

# Supplementary Figures

**Supplementary Figure 1.** Rarefaction curves of oral, sputum and fecal samples in CVID and controls. CVID, common variable immunodeficiency; dCVID, CVID with immune dysregulation; iCVID, infections-only CVID.


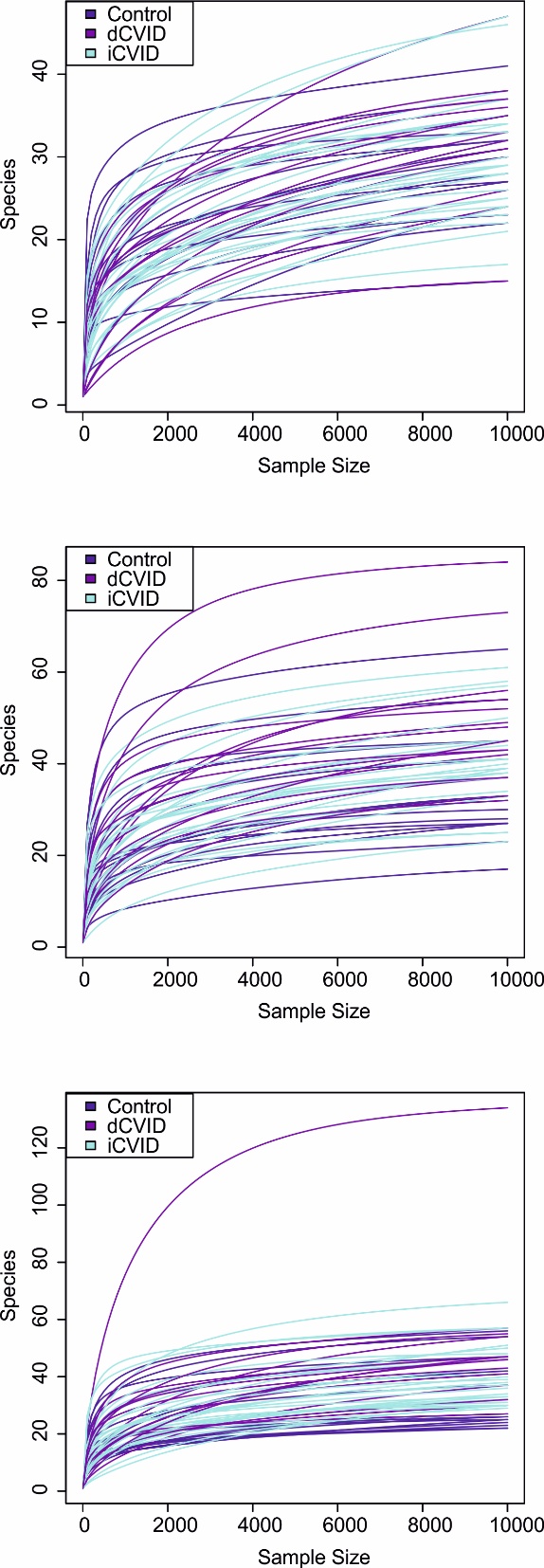


**Supplementary Figure 2:** Relative abundance of core mycobiome genera across sputum, stool, and saliva samples in dCVID, iCVID, and control groups. CVID, common variable immunodeficiency; dCVID, CVID with immune dysregulation; iCVID, infections-only CVID.


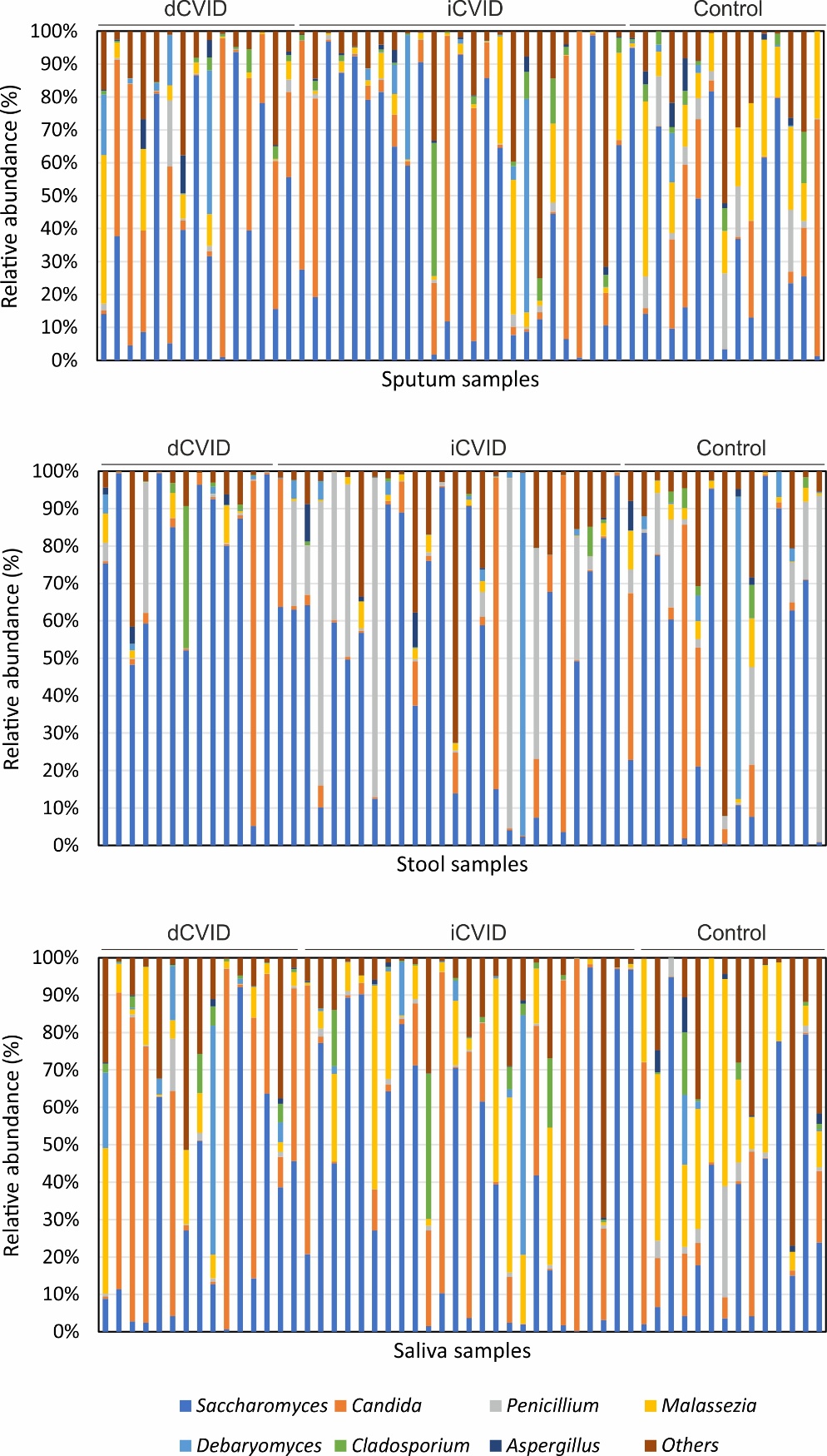


**Supplementary Figure 3.** Alpha diversity indexes in the sputum-, feces- and saliva-associated mycobiome.


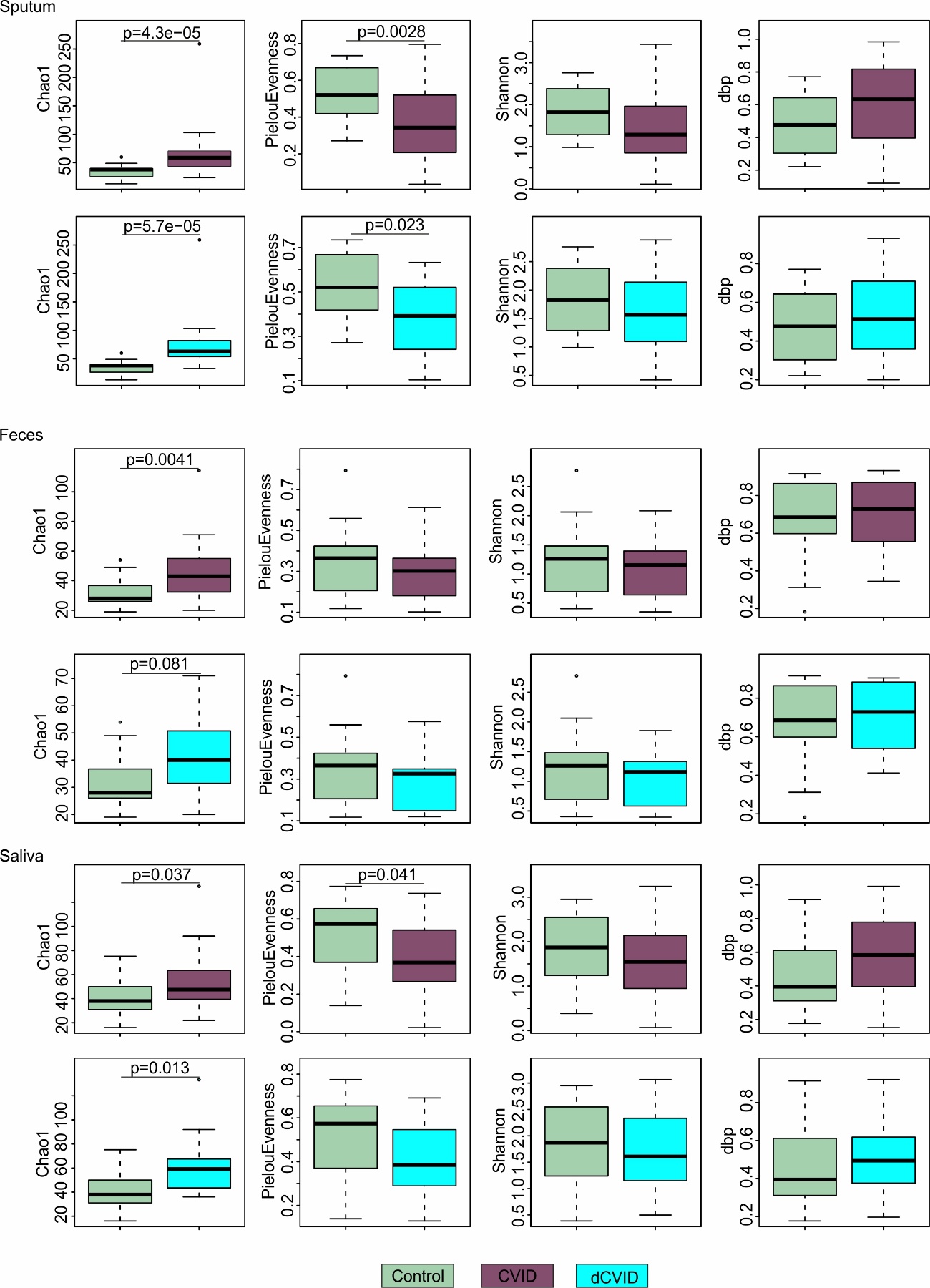


Alpha diversity indexes including Chao1, Pielou’s evenness, Shannon and dbp were calculated in the sputum-, feces- and saliva-associated mycobiome and were plotted for controls, CVID and dCVID groups. The p-value was included when the comparison was statistically significant (p<0.05).

**Supplementary Figure 4.** Correlation heatmaps between bacterial and fungal taxa in sputum samples in healthy controls and common variable immunodeficiency (CVID) patients


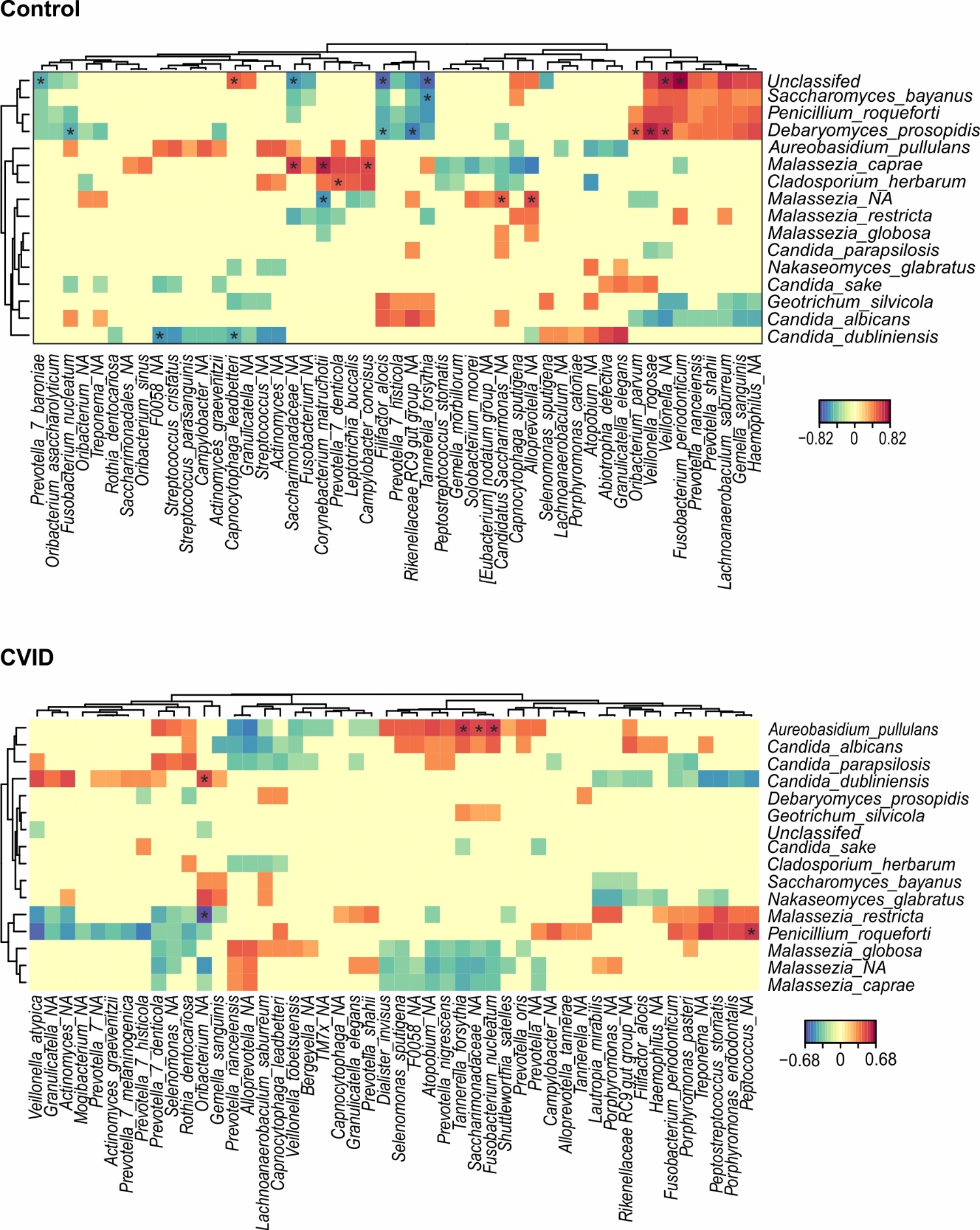


**Supplementary Figure 5.** Correlation heatmaps between bacterial and fungal taxa in stool samples in healthy controls and common variable immunodeficiency (CVID) patients


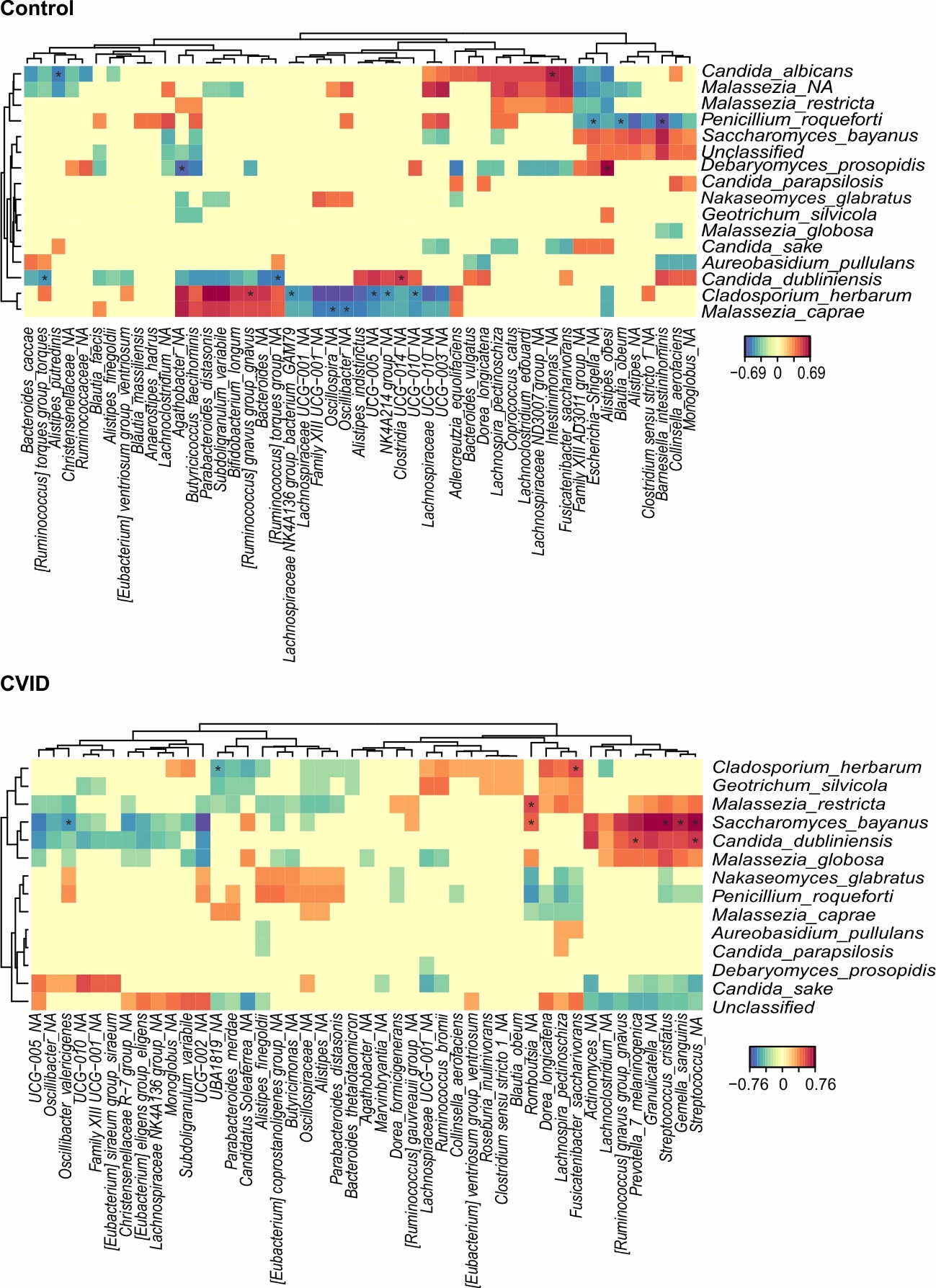


**Supplementary Figure 6.** Correlation heatmaps between bacterial and fungal taxa in saliva samples in healthy controls and common variable immunodeficiency (CVID) patients


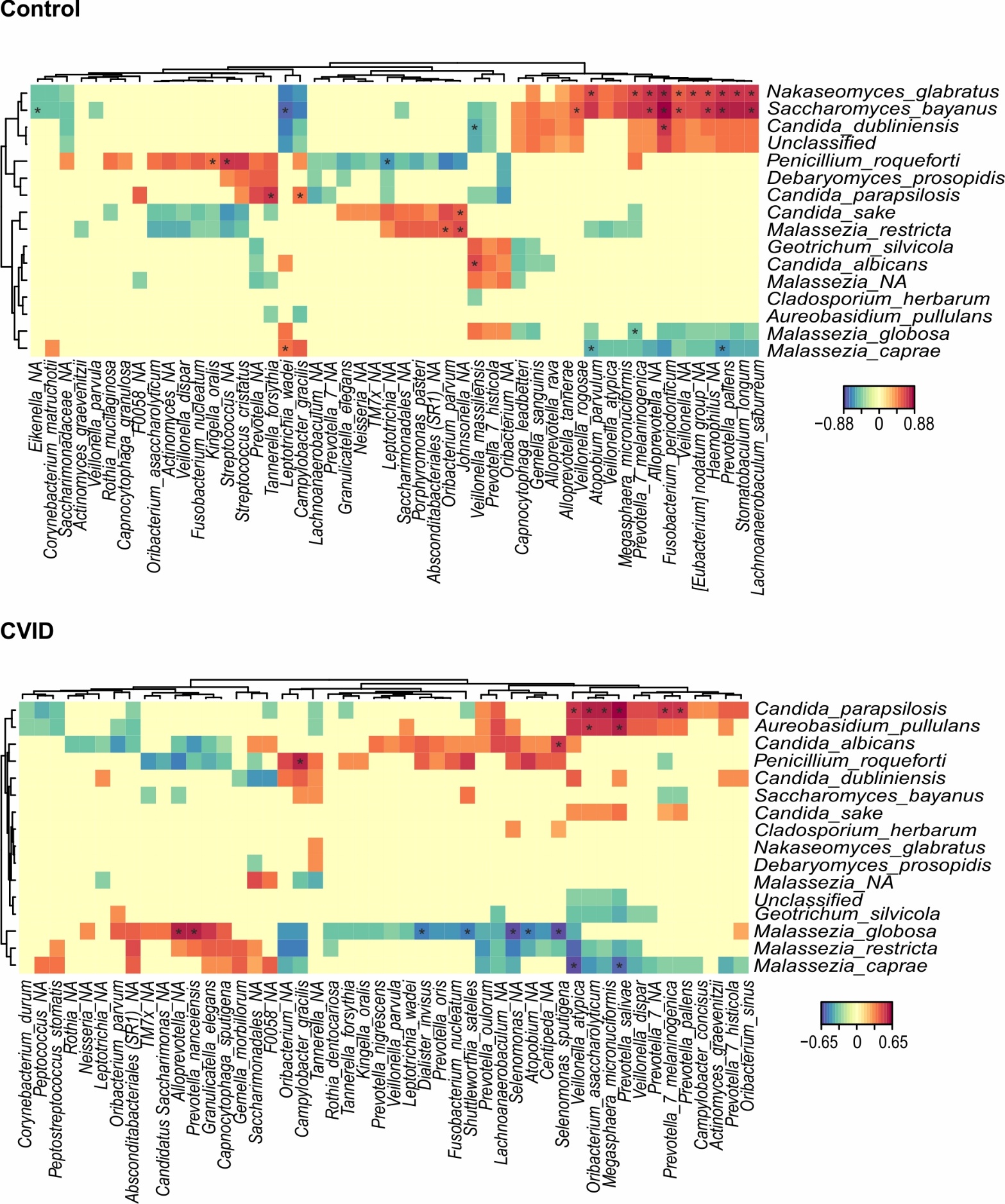

Supplement: Supplementary file 1 [file DataSheet1.docx]
